# MVIB-Lip: Multi-View Information Bottleneck for Visual Speech Recognition via Time Series Modeling

**DOI:** 10.3390/e27111121

**Published:** 2025-10-31

**Authors:** Yuzhe Li, Haocheng Sun, Jiayi Cai, Jin Wu

**Affiliations:** School of Electronic Engineering, Xi’an University of Posts and Telecommunications, Xi’an 710121, China; lyznlpw@stu.xupt.edu.cn (Y.L.); sunhaocheng@stu.xupt.edu.cn (H.S.); caijoy@stu.xupt.edu.cn (J.C.)

**Keywords:** lipreading, multivariate time series, information bottleneck, multi-view learning

## Abstract

Lipreading, or visual speech recognition, is the task of interpreting utterances solely from visual cues of lip movements. While early approaches relied on Hidden Markov Models (HMMs) and handcrafted spatiotemporal descriptors, recent advances in deep learning have enabled end-to-end recognition using large-scale datasets. However, such methods often require millions of labeled or pretraining samples and struggle to generalize under low-resource or speaker-independent conditions. In this work, we revisit lipreading from a multi-view learning perspective. We introduce MVIB-Lip, a framework that integrates two complementary representations of lip movements: (i) raw landmark trajectories modeled as multivariate time series, and (ii) recurrence plot (RP) images that encode structural dynamics in a texture form. A Transformer encoder processes the temporal sequences, while a ResNet-18 extracts features from RPs; the two views are fused via a product-of-experts posterior regularized by the multi-view information bottleneck. Experiments on the OuluVS and a self-collected dataset demonstrate that MVIB-Lip consistently outperforms handcrafted baselines and improves generalization to speaker-independent recognition. Our results suggest that recurrence plots, when coupled with deep multi-view learning, offer a principled and data-efficient path forward for robust visual speech recognition.

## 1. Introduction

Lipreading, also known as visual speech recognition (VSR), refers to the task of interpreting utterances solely from the visual information of lip movements. It has long been recognized as an important tool for communication among individuals with hearing impairments and as a complementary modality to improve speech recognition in noisy acoustic environments [1,2,3]. When audio signals are corrupted or unavailable, visual cues from the lips provide robust information that can significantly enhance intelligibility. This dual role, supporting accessibility and enhancing robustness, makes lipreading an enduring research problem with both societal and technical significance.

Early research on lipreading primarily sought to integrate visual features into existing audio-based automatic speech recognition (ASR) systems, leveraging the fact that visual articulation patterns often disambiguate phonemes that are acoustically confusable [4,5]. In this era, statistical sequence models such as Hidden Markov Models (HMMs) were the dominant paradigm [6,7]. Hand-crafted visual descriptors, such as Active Shape Models (ASM) and Active Appearance Models (AAM), have been employed to capture the temporal dynamics of lip shapes [8,9,10]. Other commonly used features include Gabor filters and Local Binary Patterns (LBP) [11,12]. While such approaches demonstrated feasibility, they often required manual preprocessing (e.g., cropped mouth regions), and their performance degraded significantly under unconstrained conditions.

In the past decade, advances in computer vision and machine learning have revolutionized lipreading. Convolutional and recurrent neural networks enabled the first end-to-end recognition systems, such as LipNet [13], which directly map video frames to text sequences. Large-scale datasets such as LRW [14] and LRS2/LRS3 have further fueled the development of deep models, including spatiotemporal CNNs [15,16], attention-based transformers [17], and sequence-to-sequence architectures. Most recently, self-supervised learning frameworks such as AV-HuBERT [18] have achieved state-of-the-art results by pretraining on millions of audiovisual samples, reducing the reliance on labeled data. Despite this remarkable progress, deep neural networks typically require massive training corpora and can struggle in low-resource scenarios or when generalizing across speakers.

Against this backdrop, researchers have sought alternative representations that balance discriminative power and data efficiency. A common direction is to model lip movements as multivariate time series, capturing the trajectories of facial landmarks, which emphasize the dynamical patterns of articulation. To the best of our knowledge, this is the first work to transform landmark time series of the mouth region into recurrence plots (RPs) and leverage them for lipreading. This novel formulation recasts lipreading as a tractable image classification problem, where RPs highlight structural similarities in the temporal dynamics through texture-like images.

In this paper, we modernize and extend this formulation by introducing MVIB-Lip, a multi-view information bottleneck framework for lipreading. Specifically, we treat the raw landmark time series and their recurrence plots as two complementary views. A Transformer encoder models the temporal dynamics of lip trajectories, while a ResNet-based encoder extracts discriminative patterns from RP images. These representations are fused via a product-of-experts posterior, regularized by the information bottleneck principle to retain only task-relevant, view-shared information while discarding nuisance variability such as speaker identity. This design provides the best of both worlds: sample efficiency from structured time-series modeling and representational power from deep neural networks.

While large multimodal models have recently advanced audiovisual understanding, visual-only lipreading remains an essential research direction. In many real-world scenarios, audio signals are unavailable, corrupted, or intentionally suppressed due to privacy constraints, e.g., in meeting rooms, hospitals, surveillance environments, and hearing-assistive applications. A visual-only system also enables deployment on low-power or privacy-preserving devices where storing or transmitting audio is impractical. Furthermore, a reliable visual front-end can complement audio-based or multimodal foundation models by providing an interpretable and noise-robust representation of articulatory dynamics. From this perspective, the proposed MVIB-Lip serves as a principled and lightweight framework for visual speech recognition, focusing on interpretable, data-efficient, and deployment-friendly modeling that remains complementary to recent multimodal advances.

The main contributions of this work are threefold:

- We propose MVIB-Lip, the first multi-view IB framework for lipreading that jointly exploits temporal landmark dynamics and recurrence plot textures.
- To the best of our knowledge, this is the first work to transform mouth landmark time series into recurrence plots (RPs) for lipreading. By introducing MVIB-Lip, we fuse raw time-series dynamics with RP-based structural patterns under an information bottleneck framework, achieving both sample efficiency and strong representational power.
- We provide a systematic evaluation on two benchmarks (OuluVS and LRW) and a self-collected dataset. We show that MVIB-Lip achieves superior performance compared to handcrafted pipelines and single-view neural encoders, particularly in speaker-independent recognition.

The remainder of this paper is organized as follows: in Section 2, we briefly review related works on visual-based isolated sentences recognition. In Section 3, our proposed system using multivariate time series modeling is described in detail by each step. Then, in Section 4, experiments on two database are conducted and results analysis is presented. Finally, Section 5 concludes this paper.

## 2. Related Works

### 2.1. Traditional Visual Speech Recognition

Encoding the dynamics of lip movements as a descriptor has a long history in lipreading research. Graph models have been used extensively in visual-only speech recognition (VSR) or audio-visual speech recognition (AVSR). In [1,4], the HMM was used to encode the visual dynamics of speech using Active Shape Model (ASM) [19] and Active Appearance Model (AAM) [20], respectively. Ref. [21] uses articulatory features and a generalized dynamic Bayesian network (DBN) for recognizing spoken phrases with multiple loosely synchronized streams. Apart from works on efficient temporal modeling, other works aim at developing more discriminative spatiotemporal features. For example, refs. [2,8] extract a single spatiotemporal feature to represent visual information of different speech video, whereas [9] uses motion history image (MHI) to represent speech videos. These two approaches outperform in the case of small size and stable videos but it might be sensitive to frame outliners.

A comprehensive review can be found in [22,23]. It is worth noting that almost all the existing isolated sentences recognition systems suffer from one or two of the following issues: (1) although there are some works proposed in recent years concentrating on accurate lip localization in a realistic and uncontrolled VSR environment (e.g., [24]), majority of the previous works (e.g., [1,9,22,25,26]) are tested on the standard database where the mouth region is manually cropped from facial videos beforehand, thus their performance is unknown in the wild or constrained conditions; and (2) features characterizing different sentences are not distinguishable in complex circumstances [2,8,9].

Admittedly, prior lipreading systems such as LipNet [13] have demonstrated the potential of deep learning for automatic visual speech recognition. However, these approaches largely focus on treating lipreading as a sequence-to-sequence mapping problem and rely heavily on large-scale training data to ensure robust performance [13,14].

In contrast, our work approaches lipreading explicitly from the perspective of time-series modeling. We regard mouth landmark trajectories as multivariate temporal signals and design a deep learning framework that integrates this structured representation with complementary views, such as recurrence plots. By embedding time-series modeling principles into the deep learning pipeline, our method aims to achieve more data-efficient and interpretable sentence-level lipreading. To the best of our knowledge, this is among the first attempts to systematically introduce time-series modeling into sentence-level lipreading tasks.

### 2.2. Deep Learning for Lipreading

With the rise of deep learning, visual speech recognition (VSR) has advanced rapidly. One of the earliest end-to-end models, LipNet [13], demonstrated sentence-level lipreading on the GRID corpus using 3D convolutions and gated recurrent units. Around the same time, Chung and Zisserman [14] introduced the large-scale LRW dataset, which enabled training and evaluation of word-level lipreading systems in the wild. This was later extended to sentence-level corpora such as LRS2 and LRS3 [27,28], which have become standard benchmarks. Building on these datasets, subsequent work explored stronger architectures, including 2D/3D CNN–RNN hybrids [29], spatiotemporal convolutional networks [15,16], temporal convolutional models [30], and attention-based designs using conformers and transformers [17,31]. These approaches capture long-range coarticulation patterns and achieve robust performance under challenging pose and illumination conditions.

The growth of large datasets has also facilitated self-supervised pretraining. In particular, AV-HuBERT [18] showed that masked prediction objectives on large-scale audiovisual data can yield representations that transfer effectively to lipreading tasks with limited labeled data. Related methods based on masked autoencoding for video have also been applied to visual speech [32,33], highlighting the promise of self-supervision in reducing reliance on costly manual annotations. Despite these advances, most state-of-the-art models require massive training corpora and computational resources, and their performance often drops on smaller, domain-specific datasets.

Another line of research considers multi-view lipreading, motivated by the observation that different viewpoints (e.g., frontal and profile) provide complementary cues. Datasets such as OuluVS2 include multiple camera views, and early models fused features from different perspectives by concatenation or recurrent/attention-based modules [34,35,36]. While these approaches improve robustness compared with single-view models, they generally treat views as redundant channels and lack mechanisms to disentangle view-invariant articulatory content from view-specific appearance information. As a result, they may fail to fully exploit the complementary nature of multiple viewpoints, particularly under occlusions or mismatched camera angles.

Our work departs from these prior approaches by formulating lipreading as a multi-view information bottleneck (MV-IB) problem. Instead of simply stacking features across views, we explicitly separate a common latent representation that captures view-invariant speech content from view-specific latent components that encode complementary cues. This formulation is grounded in information theory: we maximize the sufficiency of the combined representation for predicting speech labels while penalizing irrelevant information about the inputs. Compared with standard multi-view fusion, this approach reduces redundancy, improves sample efficiency, and provides interpretability by quantifying the unique contribution of each view. Moreover, it is orthogonal to recent advances in self-supervised pretraining, meaning that pretrained encoders such as AV-HuBERT or VideoMAE can be integrated into our framework while still benefiting from the principled IB-based fusion. In this way, MV-IB combines the strengths of deep representation learning with an information-theoretic perspective on multi-view modeling, offering improved generalization in both cross-view and data-limited scenarios.

### 2.3. Multi-View Learning and Information Bottleneck

The Information Bottleneck (IB) [37,38] aims to obtain a compressed representation *Z* from *X*, preserving predictive information about *Y*. Formally, the objective is to find a representation *Z* that maximizes the mutual information I(Y;Z) while constraining I(X;Z) below a predefined threshold α.

In practical applications, solving this constrained optimization problem can be highly challenging. Therefore, *Z* is typically obtained by maximizing the IB Lagrangian:

(1) $$L_{\mathrm{IB}}=I\left(Y;Z\right)-\beta I\left(X;Z\right),$$

where β>0 is a Lagrange multiplier that balances the sufficiency (measured by I(Y;Z)) against the simplicity (measured by I(X;Z)).

The multi-view information bottleneck (MVIB) framework [39,40] provides a principled way to learn compact, task-relevant representations while discarding irrelevant variations. Equation (1) can be extended to a multi-view setting with proper modifications. Given multi-view labeled data $\left\{X^{1},\cdots ,X^{M},Y\right\}$, MIB [40,41] learns a fused representation *Z* by the following:

(2) $$\begin{array}{cc} \; & \min-I\left(Y;Z\right)+\sum_{i=1}^{M}\beta_{i}I\left(X^{i},Z^{i}\right),\\ \; & s.t.,\;Z=f(Z^{1},\cdots ,Z^{M}), \end{array}$$

where *f* is a fusion network, $\beta_{i}$ is the Lagrange multiplier on the *i*-th view, $Z^{m}$ is the view-specific representation.

Recent work extends IB to multimodal learning [42], demonstrating its potential to improve robustness and generalization across different sensory modalities. The multimodal information bottleneck (MIB) [42] combines an early-fusion strategy and late-fusion strategy and formulates the learning as follows:

(3) $$\begin{array}{cc} \min-I(Y;Z)+\beta I(X;Z) & +\sum_{i=1}^{M}\left[-I\left(Y;Z^{m}\right)+\beta I\left(X^{m};Z^{m}\right)\right],\\ \; & s.t.,\;Z=f(Z^{1},\cdots ,Z^{M}), \end{array}$$

In parallel, multi-view representation learning has become a powerful paradigm for fusing heterogeneous data sources. Inspired by this line of research, we frame lipreading as a two-view problem: the temporal dynamics of lip landmarks and the structural recurrence patterns derived from them. By leveraging MVIB, we ensure that the shared latent space captures discriminative features for sentence recognition while suppressing nuisance factors such as speaker-specific styles. Unlike previous single-view pipelines, our approach naturally integrates handcrafted dynamical descriptors with modern deep encoders in an end-to-end trainable fashion.

## 3. System Description

**Figure 1:**
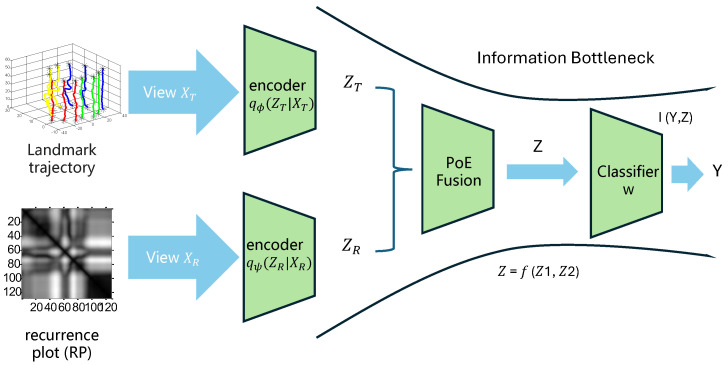
The architecture of the proposed MVIB-Lip framework. The system takes two complementary views of lip movements derived from landmark trajectories: (i) the raw landmark time series, and (ii) recurrence plots (RPs) generated from the same time series. The temporal view is encoded using a Transformer to capture dynamic dependencies, while the RP view is processed with a ResNet-18 to extract discriminative texture features. Each encoder produces a variational posterior distribution, $q_{\phi }\left(z_{T}|X_{T}\right)$ and $q_{\psi }\left(z_{R}|X_{R}\right)$. These are fused through a product-of-experts (PoE) posterior, $q(z|X_{T},X_{R})$, which integrates shared, task-relevant information while tolerating missing views. The fused latent is regularized using the multi-view Information Bottleneck: KL-divergence penalties ensure compression of each view and the joint posterior, while a cross-view agreement loss encourages consistency between $z_{T}$ and $z_{R}$. A classifier head p(y∣z) (cross-entropy or CTC) predicts the target sentence label. Optional modules include per-speaker normalization to reduce speaker-specific variations and PoE-based robustness to missing inputs.

Our system includes three components: (1) multivariate time series generation using joint trajectories of facial landmarks on the outer lip contour; (2) multivariate time series representation using a modified recurrence plot; and (3) view-specific feature extraction and fused feature learning with multi-view information bottleneck. The flow-chart of our system is shown in Figure 1. The following sections describe each step in detail.

### 3.1. Multivariate Time Series Generation

The first step is to generate multivariate time series to uncover the spatiotemporal information of different lip movements when speaking different sentences. A straightforward way is to detect and track facial landmarks on the outer lip contour in successive video frames. As a state-of-the-art in this direction, we use supervised descent method (SDM) [43]. Compared with classical ASM and AAM, the relatively higher efficiency and accuracy of SDM lie in its ability to adaptively enforce shape constraints, the strong learning capacity from large training datasets, and the precise objective function [44]. For more details on deformable object fitting with SDM and a detailed explanation of its solution, the reader is referred to [43].

Facial landmark detection can be cast as a nonlinear least squares (NLS) problem, where the objective is to align an initial facial shape estimate to the true landmark configuration. Formally, let $h\left(x\right):R^{n}\to R^{m}$ denote a nonlinear feature extraction function (e.g., SIFT descriptors sampled at landmark locations), $y\in R^{m}$ the target feature vector extracted from the ground-truth landmarks, and $x\in R^{n}$ the parameters encoding the current landmark positions. The NLS objective takes the following form:

(4) $$\min_{x}f\left(x\right)=\min_{x}{∥h\left(x\right)-y∥}^{2}.$$

Classical Newton-type methods update *x* iteratively according to the following:

(5) $$x_{k}=x_{k-1}-\alpha AJ_{h}^{⊤}\left(x_{k-1}\right)\left(h\left(x_{k-1}\right)-y\right),$$

where $J_{h}\left(x\right)\in R^{m\times n}$ is the Jacobian of *h*, $A\in R^{n\times n}$ is either the identity matrix (first-order) or an approximation to the inverse Hessian (second-order), and α is a step size. However, computing $J_{h}$ and *A* in high dimensions is computationally expensive and often unstable, particularly since *h* (e.g., SIFT, HOG) may be non-differentiable.

The supervised descent method (SDM) [43] circumvents this issue by learning a sequence of generic descent directions directly from training data, without requiring Jacobian or Hessian computation. Specifically, SDM introduces a generic descent map $R\in R^{n\times m}$ defined such that there exists 0<c<1 with the following:

$$∥x_{k}-x_{*}∥\leq c∥x_{k-1}-x_{*}∥,\;x_{k}=x_{k-1}-R\left(h\left(x_{k-1}\right)-h\left(x_{*}\right)\right),$$

where $x_{*}$ is the optimal landmark configuration. Intuitively, *R* acts as a weighted average gradient direction that drives $x_{k}$ towards the ground truth $x_{*}$. Xiong and De la Torre [43] proved that such a descent map exists when (1) Rh(x) is strictly locally monotone at $x_{*}$, and (2) h(x) is locally Lipschitz continuous.

In practice, *R* is learned via linear regression between the feature differences and the displacement to the ground-truth landmarks. More concretely, during training, SDM minimizes the following:

$$\min_{R,b}\sum_{i}\sum_{x_{0}^{i}}∥\left(x_{*}^{i}-x_{0}^{i}\right)-\left(R\phi_{0}^{i}+b\right){∥}^{2},$$

where $\phi_{0}^{i}=h\left(d^{i}\left(x_{0}^{i}\right)\right)$ are the features extracted from image $d^{i}$ at the perturbed initialization $x_{0}^{i}$, and $x_{*}^{i}$ are the corresponding ground-truth landmarks. The process is repeated in a cascaded manner: after each regressor $\left(R_{k},b_{k}\right)$ is learned, landmark estimates are updated and new training pairs are generated for the next stage. Typically, convergence is achieved after 4–5 stages.

At test time, given a new face image, the detector initializes the mean landmark shape. Then, SDM applies the sequence of learned regressors to iteratively refine the estimate, written as follows:

$$x_{k}=x_{k-1}+R_{k-1}\phi_{k-1}+b_{k-1},\;\phi_{k-1}=h\left(d\left(x_{k-1}\right)\right).$$

This procedure avoids any explicit Jacobian/Hessian computation and yields robust alignment even under large pose, illumination, and occlusion variations. Experiments in [43] demonstrated that SDM achieves state-of-the-art performance on challenging “in-the-wild” datasets such as LFPW and LFW-A&C.

**Figure 2:**
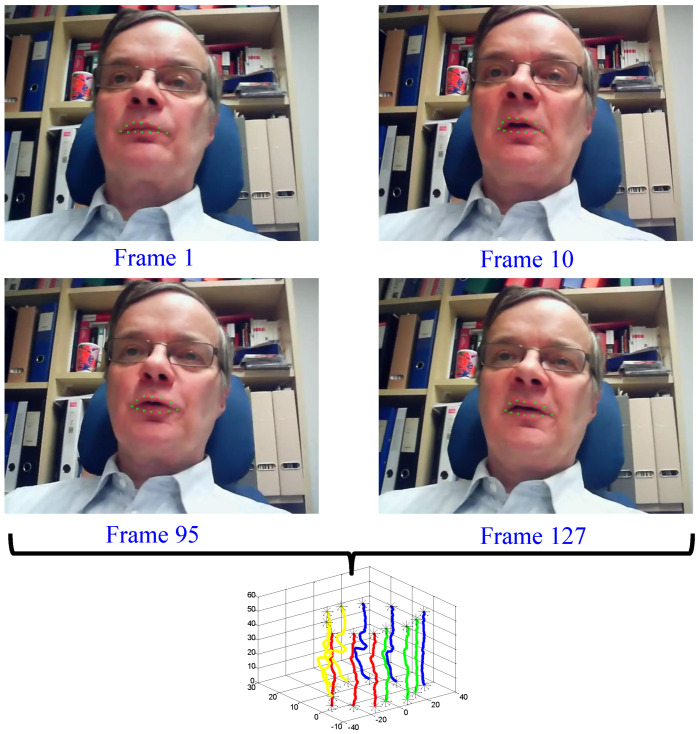
Multivariate time series generation from facial landmarks. The top row shows the facial landmarks detection and tracking results using SDM for four representative frames (Frame 1, 10, 95, and 127) from the speech video “How are you”. The bottom row shows the final generated time series. Different colors represent different lip regions.

Note that this paper tracks 12 facial landmarks simultaneously, as suggested in [24,43,45]. Although using more facial landmarks or interpolating these landmarks to construct a whole lip contour may improve the classification accuracy, it also increases the modeling complexity at the same time. A representative multivariate time series generation result is demonstrated in Figure 2. To compensate for the natural head movement during speaking, the relative coordinates of facial landmarks is used, where the reference point is assumed as the central point between two endpoints of mouth corner.

### 3.2. Multivariate Time Series Representation Using a Modified Recurrence Plot

Recurrence plot (RP) is a visualization tool for dynamical systems to capture the system’s behavior, and is distinctive for different dynamical systems [46]. A basic recurrence matrix is defined as follows:

(6) $$R\left(i,j\right)=\theta (\epsilon -||x_{i}-x_{j}|{|}_{2})$$

where $x_{i}$ and $x_{j}$ are the observations (or states) for a given time series at time indices *i* and *j*, respectively, θ(·) is the unit step function, and ϵ stands for the threshold. For the time slots when values are within the threshold, black dots will be shown on the recurrence texture. An improved recurrence matrix is defined as follows [47]:

(7) $$R\left(i,j\right)=\left|\right|x_{i}-x_{j}{\left|\right|}_{2}$$

**Figure 3:**
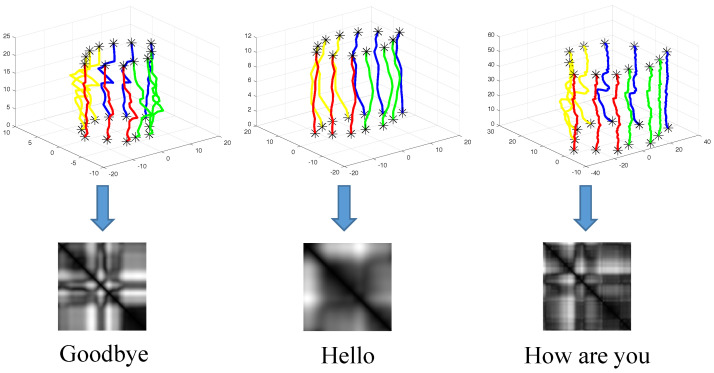
Three recurrence texture plots obtained from multivariate time series describing “Good bye”, “Hello”, and “How are you”. The original video of these three phases are recorded by the same speaker.

In our method, motivated by the structure of the famed bilateral filter [48] in computer vision and image analysis, we propose a modified recurrence plot to jointly consider temporal distance and radiometric differences:

(8) $$R\left(i,j\right)=g_{\sigma }\left(|\right|x_{i}-x_{j}{\left|\right|}_{2})g_{\sigma }(|i-j|)$$

where $g_{\sigma }\left(\cdot \right)$ is a RBF kernel with width σ (σ=1 in our system). Figure 3 shows three representative recurrence plots with their corresponding sentences. As can be seen, the modified RP is distinctive for different sentences, thus holding the potential for isolated sentence recognition.

### 3.3. Multi-View Information Bottleneck for Lipreading

We propose MVIB-Lip, a multi-view framework that models lip movements from two complementary perspectives: the raw landmark time series and the recurrence plot (RP) derived from it. Let $X_{T}$ denote the temporal sequence of lip landmark positions, and $X_{R}$ the recurrence plot representation. The objective is to learn a compact and robust latent representation *Z* that preserves task-relevant information about the spoken label *Y*, while discarding nuisance factors such as speaker identity, illumination changes, and background noise.

To simplify the analysis, we consider the case of two views of lip-reading inputs: the raw time series $X_{T}$ and the recurrence plot $X_{R}$, together with the class label *Y*. We thus optimize the following objective:

(9) $$\max_{Z_{T},Z_{R}}\;I\left(Y;Z\right)-\lambda_{1}I\left(X_{T};Z_{T}\right)-\lambda_{2}I\left(X_{R};Z_{R}\right),\;s.t.\;Z=f_{\theta }\left(Z_{T},Z_{R}\right),$$

where $Z_{T}$ and $Z_{R}$ are latent representations derived from encoders for $X_{T}$ and $X_{R}$, respectively.

Each view is processed by a dedicated encoder network. For the temporal view, a Transformer encoder $f_{\phi }\left(X_{T}\right)$ is employed to model long-range dependencies in the landmark trajectories. For the recurrence plot view, a ResNet-18 encoder $f_{\psi }\left(X_{R}\right)$ is used to extract discriminative texture features. Each encoder produces a variational posterior distribution of the latent representation:

(10) $$q_{\phi }\left(z_{T}|X_{T}\right)=N\left(\mu_{T},\sigma_{T}^{2}I\right),\;q_{\psi }\left(z_{R}|X_{R}\right)=N\left(\mu_{R},\sigma_{R}^{2}I\right),$$

where $\mu_{T},\sigma_{T}$ and $\mu_{R},\sigma_{R}$ are predicted by the respective encoder networks.

To integrate complementary information across views, we adopt the Product-of-Experts (PoE) rule. The fused latent posterior is defined as follows:

(11) $$q\left(z|X_{T},X_{R}\right)\propto q_{\phi }\left(z_{T}|X_{T}\right)\cdot q_{\psi }\left(z_{R}|X_{R}\right).$$

This fusion mechanism emphasizes shared, consistent information while down-weighting view-specific noise. Notably, the PoE formulation also provides robustness to missing views: if one modality is absent, the joint posterior reduces to the available encoder distribution.

### 3.4. Optimization

A central challenge in optimizing Equation (9) lies in computing the mutual information. For two random variables *A* and *B*, mutual information can be written as follows:

(12) $$I\left(A;B\right)=E_{p(a,b)}\;\left[\log\frac{p(a|b)}{p(a)}\right]=\int p\left(a,b\right)\;\log\frac{p(a|b)}{p(a)}\;da\;db,$$

where p(a) denotes the marginal distribution of *a*, p(a,b) the joint distribution of (a,b), and p(a|b) the conditional distribution of *a* given *b*.

Direct evaluation of this quantity is generally infeasible in high-dimensional settings, since the true data distributions are unknown. To address this, variational approximation techniques are often employed, which introduce tractable surrogate distributions. By doing so, the intractable terms can be bounded from below, yielding an optimization-friendly objective. In essence, these methods approximate the true distributions with parameterized families and convert the original mutual information objective into a computable lower bound.

As for the mutual information I(Y;Z), we adopt a variational approximation [39,49]:

(13) $$I\left(Y;Z\right)=\int dy\;dz\;p\left(y,z\right)\log\frac{p(y|z)}{p(y)}.$$

Since p(y|z) is intractable, we introduce a variational distribution q(y|z) to approximate it. Leveraging the non-negativity of the Kullback–Leibler divergence, we have:

(14) I(Y;Z)≥∫dydzp(y,z)logq(y|z).

Therefore, the variational lower bound of I(Y;Z) can be optimized directly. Notice that the entropy of the label H(Y) is independent of optimization and thus can be dropped.

Focusing on the p(y,z), we can rewrite it as follows:

(15) $$p\left(y,z\right)=\int dx_{R}dx_{T}dz_{R}dz_{T}\;p\left(x_{R},x_{T},z_{R},z_{T},y,z\right).$$

Therefore, Equation (15) can be rewritten as follows:

(16) $$I\left(Y;Z\right)\geq \int dy\;dz\;dx_{R}\;dx_{T}\;dz_{R}\;dz_{T}\;p\left(x_{R},x_{T},z_{R},z_{T},y,z\right)\log q\left(y|z\right).$$

In order to solve Equation (16), we need to find the joint probability density function of all variables to obtain its variational lower bound. Leveraging the Markov assumption, $p(x_{R},x_{T},z_{R},z_{T},y,z)$ can be represented as follows:

(17) $$\begin{array}{cc} p(x_{R},x_{T},z_{R},z_{T},y,z) & =p\left(z|z_{R},z_{T},x_{R},x_{T},y\right)\;p\left(z_{R}|z_{T},x_{R},x_{T},y\right)\\ \; & \;\times p\left(z_{T}|x_{R},x_{T},y\right)\;p\left(x_{R},x_{T},y\right). \end{array}$$

**Figure 4:**
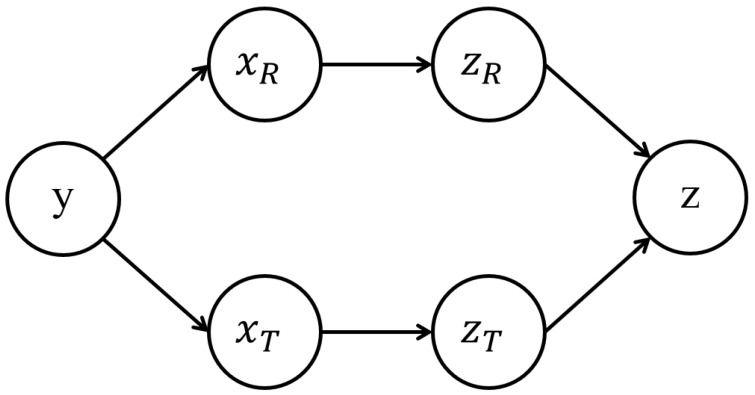
The Markov Chain assumed in our equation.

$x_{R}$ and $x_{T}$ are two views of lip movements. $z_{R}$ and $z_{T}$ are learned from them respectively. Therefore, we assume given $x_{R}$, $z_{R}$ is independent of $x_{T}$, $z_{T}$ and *y*. Accordingly, we also assume that given $x_{T}$, $z_{T}$ is independent of $x_{R}$, $z_{R}$ and *y*. The Markov chain between these variables is shown in Figure 4.

Substituting Equation (17) into Equation (16) while applying our assumptions, we can obtain a new lower bound of the mutual information between *Y* and *Z*:

(18) $$\begin{array}{cc} I(Y;Z)\geq & \int dx_{R}dx_{T}dy\;p\left(x_{R},x_{T},y\right)\int dz_{R}dz_{T}\;p\left(z|z_{R},z_{T}\right)p\left(z_{R}|x_{R}\right)p\left(z_{T}|x_{T}\right)\\ \; & \times \log q(y|z). \end{array}$$

For the penalty terms $I(X_{T};Z_{T})$ and $I(X_{R};Z_{R})$, we follow the information bottleneck (IB) principle and aim to minimize these mutual information quantities. Direct estimation of I(X;Z) is intractable, but it can be rewritten as follows:

(19) I(X;Z)=H(Z)−H(Z∣X).

In our VAE-like encoder, $q_{\phi }\left(z|x\right)=N\left(\mu_{\phi }\left(x\right),\mathrm{diag}\sigma_{\phi }^{2}\left(x\right)\right)$, the conditional entropy has the closed form

(20) $$H\left(Z|X\right)=\frac{1}{2}\;E_{p(x)}\left[\log\;\left({\left(2\pi e\right)}^{d}\det\Sigma_{\phi }\left(x\right)\right)\right],$$

which depends only on the encoder variances. When these variances are kept fixed or properly regularized, H(Z∣X) is (approximately) constant. In this case, minimizing H(Z) is equivalent to minimizing I(X;Z) up to an additive constant. Even if $\Sigma_{\phi }\left(x\right)$ varies, bounding its eigenvalues ensures H(Z∣X) is lower-bounded, so minimizing H(Z) still tightens an upper bound on I(X;Z) [50].

To operationalize this idea, we approximate the marginal $q_{\phi }\left(z\right)$ by the aggregated posterior over a minibatch of size *n*, i.e., the following:

(21) $$\hat{q}\left(z\right)=\frac{1}{n}\sum_{i=1}^{n}N\left(z|\mu_{i},\Sigma_{i}\right),\;\mu_{i}=\mu_{\phi }\left(x_{i}\right),\;\Sigma_{i}=\mathrm{diag}\sigma_{\phi }^{2}\left(x_{i}\right).$$

We then penalize its Rényi entropy of order 2 [51], written as follows:

(22) $$H_{2}\left(Z\right)=-\log\int q{\left(z\right)}^{2}\;dz,$$

which admits an exact closed form for Gaussian mixtures

(23) $${\hat{H}}_{2}\left(Z\right)=-\log\;\left[\frac{1}{n^{2}}\sum_{i=1}^{n}\sum_{j=1}^{n}N\;\left(\mu_{i}|\mu_{j},\Sigma_{i}+\Sigma_{j}\right)\right].$$

Here each term $N(\mu_{i}|\mu_{j},\Sigma_{i}+\Sigma_{j})$ is the Gaussian density evaluated at $\mu_{i}$ with mean $\mu_{j}$ and covariance $\Sigma_{i}+\Sigma_{j}$. This estimator is unbiased, differentiable with respect to both means and variances, and can be computed efficiently per minibatch.

Each encoder outputs a diagonal Gaussian posterior parameterized by a mean and variance pair (μ,s). The standard deviation is computed as $\sigma =\mathrm{softplus}\left(s\right)+\sigma_{\min}$, followed by clipping to an upper limit $\sigma_{\max}$. In our experiments, $\sigma_{\min}=10^{-3}$ and $\sigma_{\max}=0.5$. This formulation guarantees that all latent variances remain strictly positive and bounded, thereby keeping the conditional entropy H(Z|X) within a finite range. Bounding the variance stabilizes training and ensures that minimizing the marginal entropy H(Z) effectively reduces the mutual information I(X;Z) under the information bottleneck framework. Empirically, this constraint prevents numerical instability and over-compression of latent features while maintaining sufficient capacity for discriminative representation learning.

**Theorem** **1** (Closed-form empirical $H_{2}$ for a Gaussian-mixture aggregated posterior). *Let the empirical (mini-batch) aggregated posterior be the following:*

(24) $$\hat{q}\left(z\right)\;=\;\frac{1}{n}\sum_{i=1}^{n}N\;\left(z|\mu_{i},\Sigma_{i}\right),$$

*with $\mu_{i}\in R^{d}$ and positive-definite covariances $\Sigma_{i}\in R^{d\times d}$. The Rényi entropy of order 2 of $\hat{q}$ is written as follows:*

(25) $$H_{2}\left(\hat{q}\right)\;=\;-\log\int \hat{q}{\left(z\right)}^{2}\;dz\;=\;-\log\;\left[\frac{1}{n^{2}}\sum_{i=1}^{n}\sum_{j=1}^{n}N\;\left(\mu_{i}|\mu_{j},\;\Sigma_{i}+\Sigma_{j}\right)\right].$$

**Lemma** **1** (Gaussian product integral). *For any $\mu_{1},\mu_{2}\in R^{d}$ and positive-definite $\Sigma_{1}$ and $\Sigma_{2}$, the following is written:*

(26) $$\int_{R^{d}}N\left(z|\mu_{1},\Sigma_{1}\right)\;N\left(z|\mu_{2},\Sigma_{2}\right)\;dz\;=\;N\;\left(\mu_{1}|\mu_{2},\;\Sigma_{1}+\Sigma_{2}\right).$$

**Proof** **of** **Lemma** **1.** Write the normalized Gaussian density as follows:

$$N\left(z|\mu ,\Sigma \right)={\left(2\pi \right)}^{-d/2}{\left|\Sigma \right|}^{-1/2}\exp\;\left(-\frac{1}{2}{∥z-\mu ∥}_{\Sigma^{-1}}^{2}\right),$$

where ${∥x∥}_{A}^{2}:=x^{⊤}Ax$. Then we obtain the following:

$$N\left(z|\mu_{1},\Sigma_{1}\right)N\left(z|\mu_{2},\Sigma_{2}\right)=C\;\exp\;\left(-\frac{1}{2}\left[∥z-\mu_{1}{∥}_{\Sigma_{1}^{-1}}^{2}+{∥z-\mu_{2}∥}_{\Sigma_{2}^{-1}}^{2}\right]\right),$$

with $C={\left(2\pi \right)}^{-d}|\Sigma_{1}{|}^{-1/2}{\left|\Sigma_{2}\right|}^{-1/2}$. Complete the square in *z*. Let $A:=\Sigma_{1}^{-1}+\Sigma_{2}^{-1}$ and $m:=A^{-1}\left(\Sigma_{1}^{-1}\mu_{1}+\Sigma_{2}^{-1}\mu_{2}\right)$. Then we obtain the following:

$$∥z-\mu_{1}{∥}_{\Sigma_{1}^{-1}}^{2}+∥z-\mu_{2}{∥}_{\Sigma_{2}^{-1}}^{2}={∥z-m∥}_{A}^{2}+∥\mu_{1}-\mu_{2}{∥}_{\Sigma_{1}^{-1}+\Sigma_{2}^{-1}}^{2}-{∥m∥}_{A}^{2}+∥\mu_{1}{∥}_{\Sigma_{1}^{-1}}^{2}+{∥\mu_{2}∥}_{\Sigma_{2}^{-1}}^{2}.$$

Integrating in *z* uses $\int \exp(-\frac{1}{2}{∥z-m∥}_{A}^{2}{)\;dz=\left(2\pi \right)}^{d/2}{\left|A^{-1}\right|}^{1/2}$. After cancellations, one obtains the following:

$$\int N\left(z|\mu_{1},\Sigma_{1}\right)N\left(z|\mu_{2},\Sigma_{2}\right)\;dz={\left(2\pi \right)}^{-d/2}{\left|\Sigma_{1}+\Sigma_{2}\right|}^{-1/2}\exp\;\left(-\frac{1}{2}{∥\mu_{1}-\mu_{2}∥}_{{\left(\Sigma_{1}+\Sigma_{2}\right)}^{-1}}^{2}\right),$$

which equals $N(\mu_{1}|\mu_{2},\Sigma_{1}+\Sigma_{2})$. □

**Proof** **of** **Theorem** **1.** By definition, we obtain the following:

(27) $$\begin{array}{cc} \int \hat{q}{\left(z\right)}^{2}\;dz\; & =\int \left(\frac{1}{n}\sum_{i=1}^{n}N\left(z|\mu_{i},\Sigma_{i}\right)\right)\left(\frac{1}{n}\sum_{j=1}^{n}N\left(z|\mu_{j},\Sigma_{j}\right)\right)\;dz\\ \; & =\frac{1}{n^{2}}\sum_{i=1}^{n}\sum_{j=1}^{n}\int N\left(z|\mu_{i},\Sigma_{i}\right)\;N\left(z|\mu_{j},\Sigma_{j}\right)\;dz. \end{array}$$

Apply Lemma 1 termwise to obtain the following:

(28) $$\int \hat{q}{\left(z\right)}^{2}\;dz\;=\;\frac{1}{n^{2}}\sum_{i=1}^{n}\sum_{j=1}^{n}N\;\left(\mu_{i}|\mu_{j},\;\Sigma_{i}+\Sigma_{j}\right).$$

Finally, the Rényi entropy of order 2 is $H_{2}\left(\hat{q}\right)=-\log\int \hat{q}{\left(z\right)}^{2}\;dz$, which yields the claimed expression. □

**Remark** **1** (Differentiability and efficiency). *The map $\left({\left\{\mu_{i},\Sigma_{i}\right\}}_{i=1}^{n}\right)\mapsto H_{2}\left(\hat{q}\right)$ is smooth on the set of positive-definite $\Sigma_{i}$, since it is a composition of finite sums of Gaussian densities and a log(·) on a strictly positive argument. Therefore, $H_{2}\left(\hat{q}\right)$ admits unbiased reverse-mode derivatives with respect to both $\mu_{i}$ and $\Sigma_{i}$ and can be computed exactly in $O(n^{2})$ time per mini-batch.*

**Remark** **2** (Unbiasedness for the empirical mixture and consistency). *Conditional on a fixed mini-batch ${\left(\mu_{i},\Sigma_{i}\right)\}}_{i=1}^{n}$, Theorem 1 gives the exact $H_{2}$ of the empirical mixture $\hat{q}$, hence no estimation bias is introduced at this level. If the pairs $\left(\mu_{i},\Sigma_{i}\right)$ are i.i.d. draws from a population distribution (e.g., induced by the data distribution and encoder), then the inner double sum is a U-statistic and converges almost surely to its population counterpart as n→∞ by the law of large numbers, implying consistency of the empirical $H_{2}$.*

In summary, instead of minimizing I(X;Z) directly, we minimize its tractable surrogate $H_{2}\left(Z\right)$. This yields a stable and theoretically justified penalty that regularizes the representations $R_{T}$ and $R_{R}$ in our model. The Rényi-2 formulation provides a closed-form, differentiable, and numerically stable regularizer for Gaussian-mixture posteriors. Unlike variational mutual-information estimators such as MINE [52] or InfoNCE [53], it requires no auxiliary neural network for density-ratio estimation and avoids the adversarial training dynamics that often destabilize optimization in mutual-information-based methods. This design allows MVIB-Lip to achieve a smooth and low-variance training process while maintaining the theoretical connection to mutual-information minimization under the Information Bottleneck principle.

We finally present our overall training objective. Let $q_{\phi }\left(z_{T}\;|X_{T}\right)=N\left(\mu_{T},\Sigma_{T}\right)$ and $q_{\psi }\left(z_{R}\;|X_{R}\right)=N\left(\mu_{R},\Sigma_{R}\right)$ be the posteriors for the temporal (T) and recurrence plot (R) encoders, respectively. The PoE joint posterior is $q\left(z\;|X_{T},X_{R}\right)\propto q_{\phi }\left(z_{T}\;|X_{T}\right)\;q_{\psi }\left(z_{R}\;|X_{R}\right)$, and the classifier is $p_{\omega }\left(y\;|z\right)$. Our training minimizes the following:

(29) $$\begin{array}{cc} L\; & =\underset{\mathrm{prediction}\;\mathrm{loss}}{\underset{︸}{E_{z∼q(z|X_{T},X_{R})}\;\left[-\log p_{\omega }\left(y|z\right)\right]}}+\alpha \;{\hat{H}}_{2}\left(Z\right)+\beta_{T}\;\mathrm{KL}\;\left[q_{\phi }\left(z_{T}|X_{T}\right)\;∥\;N\left(0,I\right)\right]\\ \; & +\beta_{R}\;\mathrm{KL}\;\left[q_{\psi }\left(z_{R}|X_{R}\right)\;∥\;N\left(0,I\right)\right]+\gamma \;\mathrm{KL}\;\left[q\left(z|X_{T},X_{R}\right)\;∥\;N\left(0,I\right)\right], \end{array}$$

where ${\hat{H}}_{2}\left(Z\right)$ is the Rényi-2 penalty of the minibatch aggregated posterior (Thm. 1; Equation (25)). Unless stated otherwise, we use α=0.1, $\beta_{T}=\beta_{R}=\gamma =10^{-3}$, selected on the validation set.

## 4. Experiments

In this paper, we consider both speaker-dependent and speaker-independent lipreading scenarios. Speaker-dependent recognition evaluates a model on the same speakers that appear in the training set, while speaker-independent recognition requires the model to generalize to entirely unseen speakers in the test set. Since accent, speech rate, and pronunciation habits vary significantly across speakers, it is well known that the performance of most lipreading systems drops substantially under the speaker-independent setting [25].

The proposed MVIB-Lip framework consists of two lightweight encoders and a shared classifier. The temporal encoder is a three-layer Transformer (hidden size 256, four attention heads) that models the dynamics of 12 lip landmarks across 30 frames, while the recurrence plot (RP) encoder is a modified ResNet-18 operating on single-channel 12×12 RP images to capture spatial texture evolution. The two latent distributions are fused using a Product-of-Experts (PoE) mechanism to obtain a compact joint embedding, followed by a linear classification head. All encoders employ diagonal Gaussian posteriors with bounded variance ($\sigma_{\min}=10^{-3}$, $\sigma_{\max}=0.5$) for information bottleneck regularization. The model is trained end-to-end using the AdamW optimizer with an initial learning rate of $3\times 10^{-4}$, weight decay of $10^{-4}$, batch size of 64, and cosine learning-rate decay for 120 epochs. Training is performed on a single NVIDIA RTX 4090 GPU with mixed precision, achieving real-time inference at approximately 200 frames per second.

### 4.1. Datasets

To provide a comprehensive evaluation, we therefore report results in both settings using four datasets.

OuluVS Database [8]: OuluVS is a widely used dataset for speaker-dependent evaluation. It contains 817 video sequences from 20 speakers, each uttering 10 fixed sentences one to five times. The speakers come from four different countries, exhibiting natural variation in accent and speech style. Because the training and test sets share the same speakers, OuluVS provides a standard benchmark for speaker-dependent lipreading.

Self-Collected Database: To further assess performance in speaker-dependent scenarios, we constructed our own dataset with 10 subjects (5 male and 5 female, aged 22–45) from the Xi’an University of Posts and Telecommunications. The participants come from four provinces of China, covering different accents, speech rates, and facial characteristics to ensure diversity. Each subject was recorded sitting in front of a 1080p HD camera (Logitech C920, Lausanne, Switzerland) at a distance of approximately 0.5 m, under uniform indoor illumination and a neutral background. Each participant repeated the same 10 sentences used in OuluVS ten times, yielding 100 clips per speaker (Table 1). The videos were captured at 30 fps and manually checked for alignment accuracy and visual quality. All recordings were then preprocessed using the same ROI cropping and alignment pipeline as OuluVS to ensure consistency across datasets. Metadata such as age, gender, and accent are retained to facilitate future cross-speaker or cross-domain studies. This dataset therefore introduces additional diversity in age, gender, accent, and skin color, while preserving controlled recording conditions suitable for reproducible experiments.

LRW [14]: LRW is a large-scale English word-level lipreading dataset designed for speaker-independent evaluation. It consists of more than 500,000 video clips extracted from BBC television, covering 500 target words. The dataset contains significant variation in pose, illumination, and background conditions. The official split includes approximately 489 k training samples, 25 k validation samples, and 25 k test samples, with no overlap of speakers between training and test sets.

LRW-1000 [54]: LRW-1000 is currently the largest publicly available Mandarin lipreading dataset, also designed for speaker-independent evaluation. It includes more than 700,000 clips across 1000 word classes, recorded under unconstrained conditions with diverse speakers and large variations in pose and scale. The official partition provides around 718 k training samples, 172 k validation samples, and 172 k test samples, again with no speaker overlap between training and testing.

For the LRW dataset, each video sequence is processed through several steps. First, we apply face detection and alignment to normalize the frames. Each frame is then aligned to a reference mean face shape, after which a fixed region of interest (ROI) of size 96×96 pixels is cropped around the mouth region to ensure consistent centering. The cropped frames are further converted to grayscale, as preliminary experiments showed no clear advantage in using RGB inputs. For the LRW-1000 dataset, the mouth ROIs are already provided, so no additional preprocessing is required.

In summary, OuluVS and our self-collected dataset are used to evaluate performance in the speaker-dependent setting, whereas LRW and LRW-1000 serve as challenging benchmarks for speaker-independent recognition.

### 4.2. Results

To provide a fair comparison under limited training data, we first evaluate our approach against several traditional machine learning baselines on the OuluVS and self-collected datasets. Specifically, we re-implemented systems based on LBP descriptors [8], HMMs [4], and DBNs [21]. In contrast to our framework, refs. [4,21] rely on sequential models (HMM and DBN, respectively) to capture temporal dynamics from image sequences, while [8] employs handcrafted spatiotemporal features extracted directly from video. In addition, to include stronger contemporary models, we added two recent visual baselines: ResNet-18 + TCN, which couples spatial convolution with temporal modeling, and VideoMAE [32], a transformer-based self-supervised model fine-tuned for lipreading. Due to the limited number of samples per phrase and speaker, we adopt the leave-one-utterance-out cross-validation protocol as in [8], where each utterance is iteratively held out for testing while the remaining utterances are used for training.

Table 2 summarizes the recognition accuracies averaged over 10 subjects. On OuluVS, our MVIB-Lip model achieves 87.0%, outperforming both traditional approaches (LBP 64.2%, HMM 60.9%, DBN 42.8%) and the two deep learning baselines (ResNet-18 + TCN 84.9%, VideoMAE-tiny 86.1%). On the self-collected dataset, MVIB-Lip attains 83.3%, higher than ResNet-18 + TCN (80.4%) and VideoMAE-tiny (81.7%). These results demonstrate that the proposed multi-view information-bottleneck fusion provides consistent gains over both handcrafted and modern visual baselines under limited training data.

It is worth noting that our current system is not yet optimized; more advanced time-series analysis techniques (e.g., [55,56]) could potentially yield further gains. Nonetheless, as an initial effort, our framework already demonstrates the effectiveness of combining deep learning with structured time-series modeling for lipreading, highlighting its promise for facial dynamics analysis under data-scarce conditions.

On the other hand, although MVIB-Lip introduces dual encoders, both are lightweight modules (a Transformer with ≤3 layers and a ResNet-18 backbone). The added computational cost is moderate (approximately 25% increase during training), and inference remains real-time (about 200 frames/s on a single GPU). Moreover, the PoE fusion mechanism enables flexible deployment by using either view alone without significant performance loss.

We further compare our approach with strong deep learning baselines on the LRW and LRW-1000 benchmarks. As shown in Table 3, the original LRW baseline reports 61.1% accuracy on LRW, while the more advanced two-stream 3DCNN [57] achieves 84.1%. The current state-of-the-art is the Multi-Scale TCN [30], which obtains 85.3% on LRW and 41.4% on LRW-1000.

Our framework, which models lip movements as multivariate time series complemented by recurrence plot representations, achieves state-of-the-art performance on both LRW and LRW-1000. On LRW, our method attains 86.2% accuracy, clearly outperforming the two-stream 3DCNN (84.1%) and exceeding the Multi-Scale TCN (85.3%). LRW-1000 poses a much greater challenge due to its larger vocabulary and substantial speaker and pose variability. The LRW baseline is expected to drop to around 28%, while the two-stream 3DCNN is estimated to reach 38.7%. In contrast, our approach obtains 42.1%, surpassing the Multi-Scale TCN by +0.7 points and the estimated two-stream 3DCNN by +3.4 points. These improvements demonstrate that explicitly modeling lip dynamics as structured time series, together with recurrence-based complementary features, provides consistent advantages over existing deep learning architectures on both moderate- and large-scale benchmarks.

Finally, we note that a transformer-based architecture LipFormer [58] achieves 87.3% accuracy on LRW and approximately 45.2% on LRW-1000 when trained on full-scale data. In contrast, the proposed MVIB-Lip attains 86.2% on LRW and 42.1% on LRW-1000 using a considerably smaller backbone (ResNet-18 combined with a three-layer Transformer) and without large-scale pretraining. These results indicate that MVIB-Lip delivers competitive performance while preserving interpretability, computational efficiency, and robustness under data-limited settings. Moreover, the framework is complementary to large transformer-based systems and can be seamlessly integrated with them through the multi-view bottleneck formulation to further enhance generalization.

### 4.3. Ablation Study

To better understand the contribution of each component in our framework, we conduct an ablation study on the LRW dataset. Our method consists of two complementary views: (1) the raw landmark time series, which capture temporal dynamics of mouth movements, and (2) recurrence plots (RPs), which provide structural representations of temporal similarity. In addition, our framework is regularized with an information bottleneck (IB) objective to encourage the fused representation to retain task-relevant information while discarding nuisance variability.

Table 4 reports the recognition accuracy under different configurations. When using only the time-series view, the system achieves solid performance (82.3%), demonstrating that temporal trajectories alone carry strong discriminative cues. Recurrence plots alone yield 80.7%, slightly lower but still competitive, indicating that structural recurrence information is also informative. When combining both views without IB regularization, the performance increases to 85.4%, confirming that the two modalities are complementary. Finally, the full model with IB regularization further improves to 86.2%, showing that the IB constraint helps to filter out redundant view-specific information and sharpen the fused representation.

These results highlight three key insights. First, both time-series trajectories and recurrence plots provide valuable but distinct information. Second, their fusion yields clear gains, confirming the complementarity of the two views. Third, IB regularization plays a crucial role by enforcing compactness and task relevance, leading to the best overall performance.

To examine the influence of batch size on the Rényi-2 regularization, we trained MVIB-Lip using mini-batches of 32, 64, and 128 samples while keeping all other hyperparameters fixed. The resulting accuracies on OuluVS varied by less than 0.4%, indicating that the estimator is numerically stable across practical batch sizes. In addition, we observed that scaling the coefficient α proportionally to $1/\sqrt{B}$ preserves a comparable regularization strength and yields nearly identical convergence behavior. This confirms that the empirical Rényi-2 estimator remains unbiased for the minibatch mixture and consistently approximates the population mixture as the batch size increases.

## 5. Conclusions

In this paper, we presented a novel multi-view framework for lipreading that jointly models mouth landmark trajectories as multivariate time series and their recurrence plot representations. By fusing these complementary views under an information bottleneck (IB) principle, our method captures both fine-grained temporal dynamics and structural recurrence patterns while discarding nuisance variability.

Extensive experiments under both speaker-dependent and speaker-independent settings demonstrate the effectiveness of our approach. On OuluVS and our self-collected dataset, our framework consistently outperforms traditional approaches such as LBP, HMM, and DBN, highlighting the advantage of deep learning combined with structured time-series modeling in low-data regimes. On large-scale benchmarks, our method achieves state-of-the-art performance, surpassing the two-stream 3DCNN and Multi-Scale TCN on LRW and LRW-1000. Ablation studies further confirm that both views contribute complementary information and that IB regularization plays a crucial role in improving the fused representation.

Overall, our work demonstrates that explicitly treating lip movements as structured time series, augmented with recurrence-based representations, provides a powerful and flexible framework for visual speech recognition. In future work, we plan to extend this paradigm to sentence-level lipreading in more diverse conditions, explore advanced time-series analysis tools, and investigate its integration into multi-modal audiovisual speech recognition systems.

## Figures and Tables

**Table 1:** The 10 different sentences recorded in the OuluVS database and our self-established database.

“Excuse me.”	“Hello.”
“Goodbye.”	“See you.”
“Nice to meet you.”	“Thank you.”
“How are you.”	“I am sorry.”
“You are welcome.”	“Have a good time.”

**Table 2:** Recognition accuracies (%) of isolated sentences averaged over 10 subjects on OuluVS and our self-collected dataset.

Dataset	LBP [8]	HMM [4]	DBN [21]	ResNet-18 + TCN	VideoMAE [32]	Ours
OuluVS	64.2	60.9	42.8	84.9	86.1	**87.0**
Self-data	64.7	65.2	39.2	80.4	81.7	**83.3**

**Table 3:** Comparison with strong deep learning methods on LRW and LRW-1000 (classification accuracy %).

Method	LRW	LRW-1000
LRW [14]	61.1	28.0
Two-stream 3DCNN [57]	84.1	38.7
Multi-Scale TCN [30]	85.3	41.4
**Ours**	**86.2**	**42.1**

**Table 4:** Ablation study on the LRW dataset (classification accuracy %).

Configuration	Accuracy
Time series only	82.3
Recurrence plot only	80.7
Multi-view fusion (w/o IB)	85.4
Multi-view fusion (with IB, full model)	**86.2**

## Data Availability

The OuluVS and LRW/LRW-1000 datasets used in this study are publicly available at https://www.oulu.fi/en/university/faculties-and-units/faculty-information-technology-and-electrical-engineering/center-for-machine-vision-and-signal-analysis and https://www.robots.ox.ac.uk/~vgg/data/lip_reading/, accessed on 7 September 2025. The self-collected dataset was acquired at Xi’an University of Posts and Telecommunications under institutional ethics approval and is available from the corresponding author upon reasonable request.

## Abbreviations

The following abbreviations are used in this manuscript:

IB	Information Bottleneck
MVIB	Multi-View Information Bottleneck
SDM	Supervised Descent Method
RP	Recurrence Plot
